# The Osteogenic Peptide P-15 for Bone Regeneration: A Narrative Review of the Evidence for a Mechanism of Action

**DOI:** 10.3390/bioengineering11060599

**Published:** 2024-06-12

**Authors:** Cooper T. Cheng, Praveer S. Vyas, Edward James McClain, Thomáy-Claire Ayala Hoelen, Jacobus Johannes Chris Arts, Colin McLaughlin, Daniel T. Altman, Alexander K. Yu, Boyle C. Cheng

**Affiliations:** 1 Neuroscience Institute, Allegheny General Hospital, Allegheny Health Network, Pittsburgh, PA 15212, USA; cooper.cheng@ahn.org (C.T.C.); praveer.vyas@ahn.org (P.S.V.); colinpmc18@gmail.com (C.M.); 2 Department of Orthopedic Surgery and CAPHRI Research School, Maastricht University Medical Center (MUMC+), P.O. Box 616 Maastricht, The Netherlands; thomay.hoelen@mumc.nl (T.-C.A.H.); j.arts@mumc.nl (J.J.C.A.); 3 Department of Orthopaedic Surgery, Allegheny General Hospital, Allegheny Health Network, Pittsburgh, PA 15212, USA; daniel.altman@ahn.org; 4 Department of Neurosurgery, Allegheny General Hospital, Allegheny Health Network, Pittsburgh, PA 15212, USA; alexander.yu@ahn.org

**Keywords:** P-15, peptide-enhanced bone graft, bone regeneration, osteogenesis, mechanism, biomimetic peptide, orthopaedics, attachment, activation, amplification, arthrodesis

## Abstract

Bone regeneration is a complex multicellular process involving the recruitment and attachment of osteoprogenitors and their subsequent differentiation into osteoblasts that deposit extracellular matrixes. There is a growing demand for synthetic bone graft materials that can be used to augment these processes to enhance the healing of bone defects resulting from trauma, disease or surgery. P-15 is a small synthetic peptide that is identical in sequence to the cell-binding domain of type I collagen and has been extensively demonstrated in vitro and in vivo to enhance the adhesion, differentiation and proliferation of stem cells involved in bone formation. These events can be categorized into three phases: attachment, activation and amplification. This narrative review summarizes the large body of preclinical research on P-15 in terms of these phases to describe the mechanism of action by which P-15 improves bone formation. Knowledge of this mechanism of action will help to inform the use of P-15 in clinical practice as well as the development of methods of delivering P-15 that optimize clinical outcomes.

## 1. Introduction

Bone is one of the few tissues that is capable of regenerating fully following injury [1]. Regeneration in response to injury is a complex process which may be adversely impacted by numerous factors including the degree of mechanical stability, poor vascularization, underlying metabolic conditions (e.g., diabetes) and lifestyle habits such as smoking [2,3,4]. Non-union can occur in up to 48% of cases, depending upon the anatomical location and procedure complexity [5]. Although bone can heal following injury, the number of bone-related medical conditions requiring surgical intervention to achieve this is increasing. This includes, for example, resection, the implantation of endoprostheses, joint arthrodesis and fracture repair. An aging population and increased incidence of fractures (33.4% increase from 1990 to 2019 [6]) has fueled the demand for effective orthobiologics to address the ever-growing clinical need.

The surgical treatment of bone often requires the use of bone grafts to structurally and biologically facilitate osteogenesis or bone formation. Five hundred thousand bone grafting surgeries are performed in the United States alone annually [7]. As the general population ages, the demand for bone grafts may outstrip the available supply. An autograft, or autologous bone harvested often from cancellous bone of the iliac crest, is the gold standard of bone grafts [8]. While autografts are effective at promoting fusion, its routine adoption in clinical practice has been hindered by its limited supply in the body as well as the risk of donor site morbidity, including wound complication, local sensory deficit, persistent donor site pain and complications related to blood loss [9]. The regenerative capacity also diminishes with age; concentrations of growth factors and various stem cell populations in organisms are significantly reduced at older ages [10,11].

A variety of biomaterials and osteobiologics has been developed for use in bone grafting surgeries to improve healing outcomes and as a response to the supply and safety issues associated with autograft and allograft. Recent advances in bone tissue engineering have focused on using biomaterials capable of stimulating processes underlying host bone regeneration. Bone morphogenetic proteins (BMPs) are potent bone-forming agents, which led to their development for clinical use. BMPs are considered osteoinductive and are typically added onto a collagen sponge or ceramic carrier [12]. Although they have demonstrated efficacy in the formation of bone, clinical outcomes have been disputed in the literature with contradictory conclusions as to their true clinical effect. There are also safety concerns associated with high potency, delivery in high doses and lack of containment which lead to nonspecific action after migration to unintended anatomical locations. Such safety concerns include heterotopic ossification and wound complications [13,14].

Peptides, such as P-15, have been identified as a potential solution to this issue. Peptides are short-chain amino acid sequences which encode specific functions from larger protein molecules, or mimic cell binding or signaling domains. An osteogenic peptide would be advantageous compared to growth factors (e.g., rhBMP-2) due to the lower cost of production, greater simplicity of manufacturing, lower immunogenicity and the ability to be fixed at high density upon a substrate [15]. These peptide-enhanced bone grafts provide predictable cellular responses and reduce the potential for unwanted clinical effects as they have a high degree of biological specificity.

P-15 was characterized in the 1990s as a novel, synthetic 15-amino acid peptide identical to the cell-binding domain located in an alpha 1 chain of type I collagen [16]. Type I collagen, a structural protein, is the primary component of the extracellular matrix (ECM) of bone. In addition to serving as a scaffold for cells and conferring mechanical strength to tissue (in the form of collagen fibers), it plays a pivotal role in regulation of the assembly, growth and regeneration of tissue [17]. As a biomimetic peptide, P-15 has been studied extensively in the context of bone formation. Research performed in vitro demonstrates that P-15 enhances attachment, differentiation and the proliferation of cells involved in osteogenesis as well as mineralization (Table 1). Evidence of augmented osteogenesis at the cellular level is corroborated by studies using animal defect models that show that bone graft materials that contain P-15 are associated with a greater amount of bone formation and with a higher rate of arthrodesis (Table 2).

Clinical studies have demonstrated that P-15 is safe and enhances outcomes in a variety of applications including lumbar spine surgery [53], repair of periodontal bone defects [54,55] and maxillary sinus augmentation [56]. However, a step-wise summary of the mechanism of action by which P-15 augments bone formation at the cellular level has not been previously reported in the literature. Knowledge of this mechanism can help to inform the clinical use of P-15 to promote bone healing. Furthermore, an understanding of how P-15 affects osteogenesis can be utilized to develop delivery systems for this peptide that optimize patient outcomes. This narrative review provides a synthesis of the mechanism of action by which P-15 enhances bone regeneration with a special review of the key preclinical research upon which this mechanism is based.

## 2. Mechanism of P-15 in the Context of Bone Regeneration

**Figure 1:**
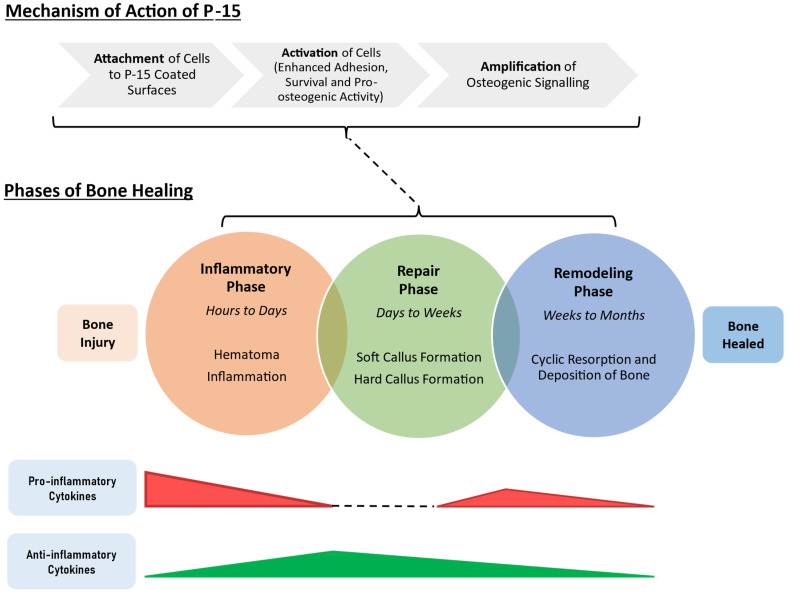
Graphical representation of the mechanism of action of P-15 in the context of the broader bone healing processes following injury. Adapted from Maruyama et al., Frontiers in Endocrinology; published by Frontiers, 2020 [57].

Bone healing occurs as the bone and surrounding tissue progress through phases of inflammation, repair and remodeling [57]. Cytokine-based cellular signaling and cell recruitment dictate transition through each phase (Figure 1). The late inflammatory phase (following the formation of a hematoma and an innate immune response) is marked by the polarization of macrophages to the M2 phenotype, followed by their secretion of osteogenic factors such as transforming growth factor β (TGFβ), which recruits osteoprogenitors to the site of injury [58]. The differentiation of osteoprogenitors into osteoblasts is stimulated by the presence of growth factors such as BMP-2 [59]. During the repair phase, the hematoma is replaced by a soft callus (unmineralized) formed by the chondrocyte secretion of cartilaginous matrix. A hard callus (mineralized) forms as chondrocytes undergo apoptosis, and osteoblastic activity results in mineral deposition. The bone tissue then enters an indefinite phase of remodeling, where it is restored and maintained through the cyclic activity of osteoblasts (which deposit bone) and osteoclasts (which resorb bone) [60].

Based on this process, key areas to target in bone healing at the cellular level include the recruitment and retention of osteoprogenitors at the site of injury, the induction of osteogenic differentiation and the proliferation and enhancement of cell signaling that promotes the deposition of matrix and bone minerals. As described in the following subsections, P-15 affects the cellular response in three main phases: attachment, activation, and amplification. Following amplification, cellular signaling determines how efficiently and effectively bone will regenerate and heal.

Numerous studies have investigated the biological activities of P-15 in vitro [16,18,20,22,26,27,33,38,41,61]. These studies employed various substrates (cell culture surfaces) to which P-15 was passively absorbed or chemically linked, including, for example, tissue culture plastic, an organic bovine bone matrix (ABM) particles, hydroxyapatite (HA) disks or titanium (Ti). Cells cultured on these surfaces included osteoblasts, osteogenic cell lines, marrow-derived mesenchymal stem cells, or periodontal ligament fibroblasts, among others. Outcomes of these studies include the viability, attachment, osteogenic differentiation and proliferation of cells, expression of markers of osteogenesis and mineralization. P-15 has been demonstrated to accelerate early bone formation in animal models, indicating the peptide can modulate the phases of healing discussed earlier [46]. The biological impact of P-15 is best described in a series of phases based upon cellular response and activity.

### 2.1. Attachment Phase

Cell attachment is the earliest phase of cell adhesion and can be mediated by physical forces such as charge–charge, Van der Waals bonding or hydrophobic interactions between cell surfaces and the substrate [62,63,64]. Cells added to P-15-coated substrates attach to the substrate in greater numbers within minutes [16,18,20,22,27,38,61], are more resistant to being rinsed away and exhibit increased viability [22,24] and exhibit a lower level of apoptosis (or programmed cell death) [24] compared to cells added to substrates lacking P-15.

However, attachment as well as stable adhesion can also be mediated by specific, high-affinity receptor–ligand interactions, e.g., between cell surface receptors like integrins and their ECM binding partner(s) on the substrate [65,66]. Integrins are cell surface receptors for ECM molecules including the collagens, and they act as a crucial mediator of cell activation [65,66,67,68]. The mechanisms underlying the enhanced cell attachment to P-15-coated surfaces are unknown yet are highly relevant to the enhanced regenerative activities characteristic of P-15-enhanced implants. Cell infiltration and attachment represent the first crucial stage of the remodeling and repair of connective tissues. Moreover, the quantity of bone formed and the quality of healing following bone injury increases with the number of viable osteoprogenitor cells [69,70]. Thus, skeletal stem cells recruited and retained locally at the bone repair site promote healing, and any cell death disrupts bone regeneration and remodeling [71].

Following attachment to a surface, cells utilize cytoskeleton dynamics to extend cell processes and adhere to the substrate [62]. During haptotaxis, the P-15 coating provides a series of attachment sites on the substrate for the cells to adhere to via lamellipodia and filopodia. This differs from chemotaxis, in which the cells must follow a soluble chemoattractant to spread, and they may thus be less likely to have a distant effect that results in heterotopic ossification (among other complications) in vivo as compared to other osteoinductive agents. Because cell survival is anchorage-dependent [72,73], P-15 initiates healing by increasing cell adhesion and survival.

### 2.2. Activation Phase

Following cell attachment, P-15 alters intracellular and extracellular signaling. While interacting with P-15, cell-substrate adhesion sites transition into focal adhesions, which anchor the cytoskeleton and plasma membrane of the cell, initiating intracellular signaling cascades [38,41]. The immunostaining of pre-osteocytes and mesenchymal cells plated on a P-15-coated surface and cultured in osteogenic differentiation media showed higher numbers of focal adhesions rich in phosphorylated focal adhesion kinase (pFAK) and α_2_ integrin compared to the control [38]. In a separate study, mesenchymal cells plated on P-15-coated tissue culture plastic and cultured in chondrogenic differentiation media developed focal adhesions rich in pFAK and α_5_β_1_ integrins compared to the control [41]. Focal adhesion kinase activity is necessary for Smad-mediated BMP signaling that results in osteogenic differentiation [74]. In general, focal adhesions that co-localize with intense pFAK and integrin staining are a hallmark of integrin-mediated cellular signaling and activation orchestrated via pFAK and associated signaling pathways [75,76]. Findings of the aforementioned studies on P-15 suggest that in addition to enhancing cell attachment and survival, exposure to this peptide results in the activation of different integrin pathways and the differentiation of cells into either osteoblasts or chondroblasts in a variable manner that is dependent on growth conditions.

### 2.3. Amplification Phase

Attachment-mediated cell activation following treatment with P-15 results in an amplification of natural signaling that is important for bone regeneration. Morphogens and cytokines important for bone healing, such as BMP-2, BMP-7 [77], and TGF-β1 [26], have been shown to be expressed in higher quantities on P-15-coated substrates [18]. TGF-β1 plays a crucial role in the induction of osteogenesis by recruiting and stimulating the proliferation of osteoprogenitors [78]. BMP-2 similarly recruits stem cells to the site of injury and serves as a potent inducer of osteogenic differentiation [79,80]. An increased expression of morphogens and cytokines following treatment with P-15 occurs within physiologically safe ranges, likely via signal amplification, as opposed to the supraphysiological concentration of BMP-2 delivered to injured bone tissue in the clinical use of rhBMP-2. Similarly, the expression of mitogenic and metabolism-influencing factors such as platelet-derived growth factor (PDGF), basic fibroblast growth factor (bFGF) and insulin-like growth factor (IGF-1) is also altered on P-15-coated substrates [33,41].

Furthermore, bone-specific genes are upregulated by cells plated on P-15-coated substrates. Markers of osteoblast maturity and mineralization have been observed at higher levels in samples with P-15 coated substrates compared to controls. These upregulated markers include alkaline phosphatase (ALP), which is required for mineralization as well as runt-related transcription factor 2 (RUNX2), which is required for bone marrow stem cell differentiation [35,37]. Within weeks to months, cultures plated on P-15 display evidence of terminal differentiation based on ECM mineralization, as evidenced by increased alizarin red staining, the genesis of bone nodules, and intense immunohistochemical staining for type I collagen [16,27,38].

## 3. Review of Select In Vitro Research on P-15

The results from the original studies conducted by Qian et al. to evaluate P-15 as a viable biomaterial suggest that as a substitute for allograft bone, P-15-coated ABM provides a good environment for bone repair (1996) [18]. These studies compared the adhesion of fibroblasts to a synthetic bone graft material formed by adsorbing P-15 onto ABM (ABM/P-15) and ABM alone. Fibroblasts adhered more strongly to ABM/P-15. In the ABM/P-15 group, sheet-like fibroblast aggregates formed, connecting bone graft material particles together. This phenomenon was not observed in ABM samples. Additionally, cells grown on ABM/P-15 exhibited significantly higher ALP activity, which was indicative of fibroblast differentiation toward an osteogenic phenotype (an early finding that P-15 is osteoinductive). Bhatnagar et al. conducted a continued study of P-15, assessing cell signaling (1999) [16]. Periodontal ligament fibroblasts (PDLF) were plated on ABM/P-15 and ABM alone, with the goal of better understanding osteoinduction. This study similarly found that ABM/P-15 promoted the structured organization of fibroblasts, development of matrix and mineralization compared to ABM alone. P-15 elicited signaling for mechanical transduction and force mediation in a manner similar to the effects of interactions between cells and collagen that increase mechanical stability [16].

Hanks and Atkinson compared the viability, apoptotic activity and morphology of cells attached to ABM/P-15 and ABM alone (2004) [24]. Human foreskin fibroblasts (HFFs) and MC3T3-E1 cells were plated on these surfaces. Cells that adhered to ABM/P-15 had a larger surface area with reduced apoptosis as compared to cells adhered to ABM alone, which tended to be smaller. Fibroblasts adhered to ABM/P-15 exhibited significantly greater viability than those adhered to ABM following serum withdrawal at each timepoint (one to four days post withdrawal). These findings indicate that exposure to P-15 results in anchorage-dependent cell survival. The authors highlight that P-15 was capable of inhibiting apoptosis even though it lacks an arginylglycylaspartic acid (RGD) motif and suggest that enhanced viability is due to changes to integrin signaling [24].

Other studies have focused on how exposure to P-15 alters cell signaling that is important for osteogenesis. Emecen et al. compared gene expression (using reverse-transcriptase polymerase chain reaction; RT-PCR) and mineralization (using Von Kossa’s method) in cells treated with and without ABM/P-15 (2009). While no differences in mineralization were observed, TGFβ and BMP-2 were upregulated in samples treated with P-15 [33]. Another gene expression study by Sollazzo et al. highlighted changes in important markers of osteogenesis using mesenchymal cells (derived from three healthy adult volunteers) treated with P-15. Gene expression was assessed using RT-PCR after seven days of treatment. Treatment with P-15 was associated with an increased activation of genes that code for osterix (an early marker of osteogenic differentiation) as well as osteopontin and osteocalcin, both of which serve as late markers of osteogenic differentiation (i.e., osteoblast maturity). As further evidence of differentiation of stem cells toward an osteogenic lineage, the expression of endoglin was found to be significant decreased with treatment with P-15. These expression profiles suggest that P-15 has the potential to induce the differentiation of human osteoprogenitors [35].

P-15 has been attached to surfaces other than ABM. Liu et al. covalently bonded P-15 to titanium alloy surfaces (Ti P-15) and cultured preostocytes (MLO-A5 cells) as well as mesenchymal cells on this surface and on tissue culture plastic as a control (2012). Preosteocytes attached significantly earlier (within one hour) on Ti P-15, exhibited better cell spreading and showed a greater number of filopodial attachments (as well as attachments that were longer) than preosteocytes on tissue culture plastic. In addition to the increase in integrin and activation of FAK signaling discussed earlier, the authors observed, in mesenchymal cells treated with P-15 as compared to control, an upregulation of early and late markers of osteogenic differentiation including RUNX2, type I collagen, osterisx, bone sialoprotein at day five and RUNX2, type I collagen and osteopontin at day 13 [38].

P-15 has also been evaluated for efficacy in promoting bone regeneration when placed in specific carriers. Hydrogels have been utilized to deliver biomaterial particles to specific areas in the human body [81]. However, hydrogels may also impede cell migration [82]. Nguyen et al. demonstrated that ABM/P-15 does not lose efficacy in promoting bone formation when suspended in hydrogels (2003). ABM/P-15 and ABM alone were suspended in sodium hyaluronate hydrogel to form ABM/P-15/Hy and ABM/Hy, respectively. In hydrogel, human osteoscarcoma cells exhibited an increased production of actin filaments and stress fibers on ABM/P-15 than on ABM alone, suggesting ABM/P-15 increased cell adhesion strength. Furthermore, HOS cells on ABM/P-15 exhibited an increase in ALP, BMP-2 and BMP-7 activity. Additionally, ABM/P-15 increased mineral deposition in HOS cells, as evaluated by Alizarin Red staining [22]. Thus, P-15 retains its osteoinductive, osteoconductive and osteogenic properties when suspended in hydrogels.

Although studies performed in vitro provide ample evidence that P-15 augments osteogenesis, they are limited by their inability to account for the diverse range of osteoblast phenotypes, life in three-dimensional environments and the complexity of biological systems.

## 4. Review of Select In Vivo Research on P-15

Studies using bone defect animal models have demonstrated that P-15 (combined with a synthetic bone graft material) enhances bone regeneration in a safe manner. Outcomes of these studies include bone volume fraction, rate of arthrodesis and frequency of adverse events.

In a study of New Zealand rabbits, Scarano et al. demonstrated the ability of P-15 to improve the healing potential of cortical bone defects (2003). Two 8 mm defects were created in each tibia. The defect space was filled with ABM/P-15 or left untreated as a control. Almost all defects treated with ABM/P-15 were found to be filled with newly formed bone. A majority of the P-15 particles found within the newly formed bone did not show signs of resorption and were almost completely surrounded by mature bone. In comparison, a limited quantity of newly formed bone was observed in untreated defects [45]. A similar study conducted by Lindley et al. found that P-15 significantly increased new bone formation in a long bone defect leporine model (2010). Tibial and femoral defects filled with the P-15 construct had more bony in-growth at two, four and eight weeks post-surgery than untreated defects [83].

Pedersen et al. compared tibial defects filled with ABM/P-15 to untreated defects in normal and osteoporotic Norwegian brown rats, using a paired study design (2015). Histomorphometry showed significantly greater bone volume formation in osteoporotic rats treated with ABM/P-15 but no significant difference between the groups in normal rats. Despite this, this study provides evidence that P-15 is capable of enhancing bone regeneration in compromised environments where repair is historically difficult [50].

Accelerated bone formation with P-15 has been observed in various large animal models as well. Thorwarth et al. compared ABM/P-15, ABM alone and autologous bone as bone graft materials using a porcine skull defect model (2005). A porcine model was selected based on the comparability of bone-healing characteristics and circulation of blood through tissue between humans and pigs. Treatment with ABM/P-15 resulted in accelerated bone growth as compared to ABM with histological evaluation showing mineral deposition at three days (bone formation was observed at this timepoint for autologous bone as well). Microradiographic imaging of mineralization showed significantly greater mineralization for ABM/P-15 than for ABM at 12 weeks; however, at six months, mineralization was comparable between the two groups. Bone growth was greatest for the autologous graft. It is important to note that calvarial bones differ physiologically and anatomically from long bones. Nonetheless, this study suggests that P-15 is osteoinductive in large animals [46].

Ding et al. evaluated the efficacy of P-15 in an ovine long bone model (2014) [49]. In this model, implants were inserted into the distal femoral condyles bilaterally with one implant in medial condyle and one in lateral condyle. A concentric gap of 3.5 mm was left between the implant and host bone. This gap was filled with ABM/P-15 as well as several alternate bone graft biomaterials. ABM/P-15-filled gaps had thicker bone growth than allograft-filled gaps. The competitive groups had a higher surface area/volume ratio than those filled with ABM/P-15. These data indicate that the increased bone density of ABM/P-15 was attributed to P-15’s enhanced osteogenic properties and not the biomaterial’s particle size. The ABM/P-15 cohort was also observed to possess similar mechanical and osteoconductive properties to allografts. The results of the study suggest ABM/P-15 is a viable alternative to allografts for the purpose of healing a long bone defect.

Multiple studies utilizing ovine spine models have been conducted to assess whether ABM/P-15 is effective in promoting spinal fusion. Sherman et al. randomized ewes to undergo anterior–lateral interbody fusion at the L3/L4 and L4/L5 levels with PEEK interbody devices with the device in one level packed with autologous bone and the other packed with ABM/P-15 in a randomized fashion (2010). At six months, there were no differences between autologous graft and ABM/P-15 with regard to micro-CT and histomorphometric endpoints [48]. In a study by Axelsen et al., sheep underwent posterior lumbar fusion without instrumentation for fixation (2019). It should be noted that this is a challenging model for the evaluation of bone grafts due to the lack of stability and graft containment. Each sheep received both ABM/P-15 and allograft; graft treatment was randomized across two levels (L2/3 and L4/5). After 4.5 months, both groups were evaluated with a micro-CT scanner and histology to enable qualitative histology with 2D sections and 3D reconstruction images used to assess success of fusion. The fusion rate for sheep treated with ABM/P-15 (37%) was lower than that for sheep treated with allograft (68%) [51]. One reason for this finding may be the challenging PLF model that was used. These findings indicate that the use of an interbody device or other implant which helps to contain the graft material may improve outcomes. This study also demonstrates that the risk of heterotopic bone formation is low, as despite migration of the material, no bone formation was observed. The mechanism of P15 facilitating bone formation may therefore be context dependent.

More recently, Loenen et al. utilized an ovine lumbar interbody fusion model to compare ABM/P-15 to autografts harvested from the iliac crest (2022). Thirty sheep were randomized to treatment. After three months, sheep treated with ABM/P-15 exhibited significantly more mature, denser and thicker trabecular bone structures. After six months, the P-15 and autograft groups exhibited similar bone growth outcomes, indicating that P-15 can expedite bone formation without any evidence of adverse effects on systemic health [52].

## 5. Discussion

P-15 has been widely studied in humans in a variety of clinical applications, such as long bone fracture healing, spinal fusion, and periodontal bone healing. While preclinical research provided the evidence to support studies of P-15 in humans, findings from clinical studies further warrant a better understanding of the mechanism by which P-15 works, for example, drug delivery. These articles apply the concepts discussed in these preclinical papers to clinical applications. In a pilot trial evaluating the effects of P15-BGM (P-15/ABM suspended in hydrogel), Gomar et al. evaluated the efficacy of P-15 healing in human long bone. Testing occurred in two groups of patients with long bone delayed union and nonunion fractures. Callus formed by P15-BGM did not appear to significantly differ from naturally formed callus. Trabecular structures were observed to have formed in the newly formed bone. Resorbed P15-BGM particles were observed to be surrounded by newly formed bone directly attached to the particles, indicating P15-BGM enhanced osteoinduction and osteoconduction. The results of this study suggest P15-BGM is a viable treatment option for healing long bone fractures [84].

In a clinical trial evaluating the effects of i-Factor (commercialized ABM/P-15), Arnold et al. demonstrated that i-Factor is an effective treatment option for use in anterior cervical discectomy and fusion (ACDF) [85]. An allograft ring containing either i-Factor or autograft tissue was inserted into the disk space between either the C5–C6 or C6–C7 vertebrae. An anterior cervical plate was then placed to span the disk space. Subjects in the i-Factor test group had a higher successful fusion rate, higher reduction in disability in daily life, higher neurological success, and similar rate of adverse events to subjects in the autograft test group. I-Factor subjects had a higher rate of overall success, which is characterized by success in all four of the FDA-mandated success criteria. Subjects in the i-Factor test group had similar success rates to subjects in the autograft test group in secondary criteria, which include VAS pain quantification scores in the neck and arm/shoulder, patient-reported physical and mental operation effects, and Odom criteria [85]. The results of this study suggest i-Factor is a safe and effective treatment option for use in spinal fusion procedures.

In the early published articles within the periodontal literature, Krauser et al. discuss a case study of PepGen P-15 use with similar results. The device including PepGen P-15 was implanted in a patient undergoing a maxillary sinus elevation procedure. The authors describe a mechanism of action including cell attachment, angiogenesis, cell differentiation (activation), induction, and finally bone regeneration (amplification). The preliminary understanding of the distinct phases of bone healing by the authors have been refined to the three-phase mechanism of action that now describes the mechanism of bone healing attributed to ABM/P-15. The conclusions the authors were able to derive from the case study included an enhanced bone regeneration in the maxillary sinus graft model without autograft and in half the time traditionally required. The preliminary results of the Krauser case study is consistent with the periodontal studies that have incorporated P-15 in the protocol [86].

P-15 has been shown to improve bone formation outcomes in animal models and in humans. Preclinical evidence suggests that bone regeneration and maturation in the presence of P-15 is enhanced by stimulating the adhesion of pre-osteoblasts and osteoblasts and directly increasing cell number and viability, and by subsequently amplifying downstream cellular activity through their enhanced secretion of mitogens, cytokines and morphogens, which should have far-reaching effects within the fusion site and at its margins. As P-15 mimics the cell binding domain of type I collagen, these effects have been demonstrated in numerous preclinical models and appear ubiquitous to all bone-healing environments. The control groups that were chosen in these studies allow for a clear understanding of the osteoinductive effects of P-15 in vitro, and in the case of studies performed in vivo comparing ABM/P-15 to autograft, they set a high standard for the efficacy of this peptide for use in bone repair. Many of the studies discussed in this review also overlap with regard to findings related to enhanced cell attachment and amplified osteogenic signaling following exposure to P-15. We thus conclude that at the cellular level, the specific activities of P-15 can be classified into the phases attachment, activation and amplification.

## Figures and Tables

**Table 1:** List of studies performed in vitro on P-15.

	Authors and Year (See References for Complete Citations)	Cell Line	Study Groups	Key Results	Supporting Evidence for Mechanism Phase		
					Initiation	Activation	Amplification
**1**	Qian and Bhatnagar, 1996 [18]	Human dermal fibroblasts	Bovine ABM with a variable amount of P-15 coating	Fibroblast attachment increased with amount of P-15 on the surface of the ABM particles. Cells on ABM/P-15 formed three-dimensional structures and exhibited ALP activity.	X	X	
**2**	Bhatnagar et al., 1997 [19]	Human dermal fibroblasts from foreskin	Cells treated with varying concentrations of P-15	P-15 inhibited the binding of fibroblasts to collage, likely due to saturation of collagen binding sites on the cells by P-15.	X		
**3**	Bhatnagar et al., 1999 [16]	Human periodontal ligament fibroblasts	(A) ABM (B) ABM/P-15	More cells associated with ABM/P-15 than with ABM. Cells grown on ABM/P-15 formed complex three-dimensional structures, whereas cells grown on ABM formed simple monolayers. Mineralization was observed for ABM/P-15 samples.	X		X
**4**	Lallier et al., 2001 [20]	Human periodontal ligament fibroblasts	Cells plated on a variety of bone graft materials including non-demineralized freeze-dried bone, demineralized freeze-dried bone, ABM, ABM/P-15, Bio-Oss, and Osteogen	Increased rate of cell attachment was observed for ABM/P-15 as opposed to other graft materials.	X		
**5**	Lallier et al., 2003 [21]	Gingival and dermal fibroblasts	Cells plated on (A) Freeze-dried bone (B) HA (C) HA/P-15 (D) Low-density HA.	Cells attached more strongly to bone than to HA-based grafts. There were no differences in fibroblast attachment between HA and HA/P-15. Addition of P-15 to dental root shavings enhanced fibroblast attachment. P-15 did not have an effect on proliferation of cells on root shavings.	X		
**6**	Nguyen et al., 2003 [22]	Human osteosarcoma cells	(A) ABM/Hy (B) ABM/P-15/Hy	HOS cells on ABM/P-15/Hy expressed ALP and BMP at higher levels and deposited more mineralized bone matrix.	X	X	X
**7**	Carinci et al., 2004 [23]	MG-63	(A) Without P-15 (B) With P-15	Genes that were differentially expressed between P-15 exposure conditions are involved in cell signaling, differentiation and apoptosis.		X	X
**8**	Hanks and Atkinson, 2004 [24]	Human foreskin fibroblasts	(A) ABM (B) ABM/P-15	Viability, level of apoptosis, and viable cell attachment were significantly improved for the ABM with P-15 group.	X		
**9**	Kübler et al., 2004 [25]	Human osteoblasts derived from iliac cancellous bone	(A) Control (B) Phytogene HA (C) α-tricalcium phosphate (D) Low-temperature bovine HA (E) High-temperature bovine HA (F) High-temperature bovine HA enhanced with P-15	Proliferation and viability (as measured by WST-1) and differentiation were highest for cells treated with high-temperature bovine hydroxyapatite enhanced with P-15. The P-15 based graft material was the only tested material to result in the formation of highly dense collagen fibers. This graft material also exhibited strong attachment to cells compared to other materials.	X		X
**10**	Trasatti et al., 2004 [26]	Rat osteoblasts (primary culture)	(A) BioOss (B) OsteoGraf N-300 (C) PepGen P-15	TGF-β1 was produced at significantly higher levels in cells treated with ABM/P-15 as compared to BioOss and OsteoGraf N-300. Mineralization occurred with all graft materials.		X	X
**11**	Yang et al., 2004 [27]	Human bone marrow stromal cells	ABM: (A) Without P-15 (B) With P-15 (in conditions promoting either basal or osteogenic differentiation)	P-15 increased ALP activity and expression of BMP-2 after one and five days. Cells treated with P-15 exhibited attachment, spreading and ALP-specific activity. Other findings for P-15 included mineralization, cell ingrowth and formation of bridged three-dimensional cellular structures.	X		X
**12**	Turhani et al., 2005 [28]	Human osteoblasts	Cells cultured on: (A) HA calcified from red algae (B) Deproteinized bovine HA (C) Bovine HA carrying P-15 (D) Tissue-culture polystyrene	Cells cultured on bovine HA carrying P-15 exhibited a continuous increase in DNA content and protein synthesis (a surrogate for proliferation) and increased ALP activity. Osteogenic differentiation was further evidenced by expression of genes encoding proteins like osteocalcin and osteopontin.		X	X
**13**	Dereka et al., 2006 [29]	Human periodontal ligament cells	bFGF with (A) cortical and cancellous allograft (B) ABM/P-15	Cells treated with ABM/P-15 exhibited significantly increased proliferation.	X	X	
**14**	Palmieri et al., 2007 [30]	Osteoblasts	(A) PerioGlas (silicate-based) (B) P-15	Both silicate-based synthetic bone and P-15 enhance the translation of several miRNA associated with osteogenic genes.		X	
**15**	Yuan et al., 2007 [31]	Periodontal ligament fibroblasts	ABM: (A) Without P-15 (B) With P-15	Viability and osteogenic activity were higher for cells cultured on ABM/P-15 as compared to cells cultured on ABM alone. Annexin II, a protein important for cell movement and cytoskeleton function, bound to ABM/P-15 better than it did to ABM alone. Addition of an anti-Annexin II antibody decreased osteogenic activity in cells exposed to P-15, potentially implicating this protein in interactions between fibroblasts and P-15.	X	X	
**16**	Palmieri et al., 2008 [32]	MG-63	(A) Without P-15 (B) With P-15	P-15 alters transcription in osteoblast-like cells.		X	
**17**	Emecen et al., 2009 [33]	Human periodontal ligament cells	ABM: (A) Without P-15 (B) With P-15	ABM/P-15 increased cell proliferation and expression of TGF-β and BMP-2 as compared to the control on days three and seven. Expression of IGF-I and b-FGF was decreased, and expression of PDGF was increased, on day three in cells treated with ABM/P-15.		X	X
**18**	Herten et al., 2009 [34]	Human primary osteoblasts Bone marrow mesenchymal stem cells Nonadherent myelomonocytic cells	(A) Polystyrol (B) Bio-Oss Spongiosa (C) Tutodent Chips (D) PepGen P-15 (E) Ostim (F) BioBase (G) Cerasorb	Cell viability factor was high for P-15.	X		X
**19**	Sollazzo et al., 2010 [35]	Human bone marrow stem cells	(A) Without P-15 (B) With P-15	Upregulation of transcriptional factors including SP7 and bone-related genes including COL1A1 and ALPL was observed in cells incubated with P-15. Downregulation of endoglin, a mesenchymal stem cell marker, in cells incubated with P-15 serves as evidence that P-15 promotes the differentiation of osteoprogenitors.		X	
**20**	Vordemvenne et al., 2011 [36]	Osteoblast-like cell cultures	(A) Platelet-derived growth factor (PDGF) (B) P-15 (C) TP508 (D) PDFG/P-15 (E) PDGF/TP508 (F) PDGF/AC-100	P-15 and PDGF together decrease time to onset of calcification in osteoblasts as compared to PDGF alone.			X
**21**	Lauritano et al., 2012 [37]	Bone marrow derived stem cells Primary human osteoblasts	(A) Without P-15 (B) With P-15	Genes including RUNX2, ALPL, FOSL1 were upregulated in cells treated with P-15. Most genes assessed in this study were differentially expressed between cell lines at day 30.		X	X
**22**	Liu et al. 2012 [38]	Osteoblasts Mesenchymal cells	Titanium (Ti): (A) Without P-15 (B) Covalently bonded to P-15	Cell attachment and proliferation were enhanced on Ti/P-15 as compared to Ti alone. Cells grown on Ti/P-15 also exhibited increased filapodial attachment, expression of α_2_ integrins, integrin signaling and expression of osteogenic differentiation markers.	X	X	
**23**	Pereira et al., 2013 [39]	Rat calvarial osteogenic cells	Ti and calcium phosphate with: (A) P-15 low dose (20 µg/mL) (B) P-15 high dose (200 µg/mL)	Low and high dose surfaces all equally supported changes in the mRNA expression profile of key osteoblast markers, ultimately resulting in enhanced ECM mineralization	X	X	X
**24**	Cheng et al., 2016 [40]	Human jawbone mesenchymal stem cells	Cells incubated with varying concentration of P-15	Stiffness increased with increasing concentration of P-15. P-15 increased the adhesion energy between cells and hydroxyapatite. Morphological changes between cells cultured with P-15 and those not cultured with P-15 may explain differences in adhesion between groups.		X	
**25**	Zhang et al., 2017 [41]	Mouse mesenchymal cells cultured in chondrogenic conditions	(A) Without P-15 (B) With P-15	Increased chondrogenesis and activation of α_5_ integrin were observed with P-15 treatment as compared to the control. This does not appear to be mediated by direct integrin binding but rather through promotion of integrin signaling.	X	X	
**26**	Fu et al., 2019 [42]	Osteoblasts	Cells grown on: (A) Ti (B) Ti/P-15 (C) Polymer-grafted Ti (D) Polymer-grafted Ti/P-15	Polymer-grafted Ti discs with P-15 exhibited improved osteoblast attachment and mineral deposition (as compared to Ti and Ti with P-15 adsorbed to it in a simple manner).	X		X
**27**	Mohanram et al., 2020 [43]	Human bone marrow mesenchymal stem cells (HBMSC) Human dental pulp stem cells (HDPSC)	(A) ABM and HBMSC (B) ABM and HDPSC (C) ABM/P-15 and HBMSC (D) ABM/P-15 and HDPSC	ABM-P-15 promoted osteogenic differentiation and bone matrix formation both in vitro and in vivo.			X
**28**	Wang et al., 2022 [44]	Rat bone marrow mesenchymal stem cells	(A)–(C) Polylactic acid glycolic acid scaffolds containing a hydrogel consisting of equal amounts of P-15 and BMP-9 (concentrations of 0%, 2% and 4%) (D) PLGA scaffold without hydrogel	ALP, type I collagen, osteocalcin, RUNX2 and Sp7 were upregulated in cells incubated with PLGA with hydrogel containing P-15 and BMP-9.		X	X

**Table 2:** List of select studies performed in vivo on P-15.

Authors and Year (See References for Complete Citations)	Animal Model Type (*n*)	Model Description (*n*)	Study Groups	Timepoint	Summary of Results
Scarano et al., 2003 [45]	Leporine (5)	Defects on each tibia	Defects were filled with ABM/P-15 (with or without hydrogel carrier) or were left untreated as a control	4 weeks	Treatment with ABM/P-15 with and without a hydrogel carrier resulted in significantly greater new bone formation in the cortical drilled defects than in the control and did not engender the presence of inflammatory infiltrate cells.
Thorwarth et al., 2005 [46]	Porcine (24)	Bone defects of the porcine skull	Defects were treated with ABM grafts and ABM grafts carrying P-15	6 months total with 8 examinations	ABM/P-15 accelerated mineralization starting at day 3 in a large animal model.
Sarahrudi et al., 2008 [47]	Leporine (24)	5 mm segmental bone defects	Defects were treated with ABM/P-15 or left untreated	4, 8, and 12 weeks	No enhanced or accelerated growth was observed in this long bone critical-size defect model.
Sherman et al., 2010 [48]	Ovine (6)	Lumbar interbody fusion	PEEK rings at one level were filled with autograft and PEEK rings at the other level were filled with ABM/P-15	3 months and 6 months CT scans; histomorphometry analysis at 6 months	Fusion rate did not differ between ABM/P-15 and autograft. There were no differences in any imaging or histomorphometric findings at 6 months.
Ding et al., 2015 [49]	Ovine (8)	Critical size defects in the distal femoral condyles	Defects were treated with: (A) allograft (B) ABM/P-15 (C) hydroxyapatite (HA) + βtricalciumphosphate (βTCP) + Poly-Lactic-Acid or (D) ABM/P-15 + HA/βTCP-PDLLA	9 weeks	Defects treated with ABM/P-15 exhibited significantly greater tissue volume fraction and thickness (microarchitecture). ABM/P-15 performed at least as well as allograft with regard to bone formation.
Pedersen et al., 2015 [50]	Rodent (24)	Bilateral proximal tibia defects	Rats were divided into normal and osteoporotic groups; defects were treated with ABM/P-15 or left untreated	Post-op day 0, 14, and 21	Defects in osteoporotic rats treated with ABM/P-15 exhibited increased bone formation as compared to the control.
Axelsen et al., 2019 [51]	Ovine (12)	Posterolateral lumbar fusion	One level was treated with an ABM graft, and the other level was treated with an ABM/P-15 graft	4.5 months	P-15 was associated with a reduced rate of fusion at 4.5 months. Containment of the graft material may improve outcomes. Despite migration, no heterotopic ossification was observed.
Loenen et al., 2022 [52]	Ovine (30)	Lumbar interbody fusion	Half of the sheep received autograft and half received a P-15 based graft substitute	1, 3, and 6 months	At 3 months, one formed in sheep treated with the P-15-based graft was significantly denser and stiffer than that in sheep treated with autograft. After 6 months, P-15 and autograft showed similar fusion results. Treatment with P-15 did not result in any adverse events.
